# Prevalence, Characterization, and Mycotoxin Production Ability of *Fusarium* Species on Korean Adlay (*Coix lacrymal-jobi* L.) Seeds

**DOI:** 10.3390/toxins8110310

**Published:** 2016-10-27

**Authors:** Tae Jin An, Kyu Seop Shin, Narayan Chandra Paul, Young Guk Kim, Seon Woo Cha, Yuseok Moon, Seung Hun Yu, Sang-Keun Oh

**Affiliations:** 1 Department of Herbal Crop Research, National Institute of Horticultural and Herbal Science (NIHHS), Eumseong, Chungbuk 27709, Korea; atj0083@korea.kr (T.J.A.); ncpaul@korea.kr (N.C.P.); kimyguk@korea.kr (Y.G.K.); insam@korea.kr (S.W.C.); 2 Department of Applied Biology, College of Agriculture & Life Sciences, Chungnam National University, Daejeon 34134, Korea; seopbee@korea.kr (K.S.S.); shunyu@cnu.ac.kr (S.H.Y.); 3 Bioenergy Crop Research Institute, National Institute of Crop Science, Rural Development Administration, Muan 58545, Korea; 4 Laboratory of Mucosal Exposome and Biomodulation, Department of Biomedical Sciences, Pusan National University School of Medicine, Yangsan 50612, Korea; moon@pusan.ac.kr

**Keywords:** adlay seeds, ELISA, *Fusarium*, morphological data analysis, mycotoxins, phylogenetic analysis

## Abstract

Adlay seed samples were collected from three adlay growing regions (Yeoncheon, Hwasun, and Eumseong region) in Korea during 2012. Among all the samples collected, 400 seeds were tested for fungal occurrence by standard blotter and test tube agar methods and different taxonomic groups of fungal genera were detected. The most predominant fungal genera encountered were *Fusarium*, *Phoma*, *Alternaria*, *Cladosporium*, *Curvularia*, *Cochliobolus* and *Leptosphaerulina*. *Fusarium* species accounted for 45.6% of all species found; and, with phylogenetic analysis based on the combined sequences of two protein coding genes (EF-1α and β-tubulin), 10 *Fusarium* species were characterized namely, *F. incarnatum* (11.67%), *F. kyushuense* (10.33%), *F. fujikuroi* (8.67%), *F. concentricum* (6.00%), *F. asiaticum* (5.67%), *F. graminearum* (1.67%), *F. miscanthi* (0.67%), *F. polyphialidicum* (0.33%), *F. armeniacum* (0.33%), and *F. thapsinum* (0.33%). The *Fusarium* species were then examined for their morphological characteristics to confirm their identity. Morphological observations of the species correlated well with and confirmed their molecular identification. The ability of these isolates to produce the mycotoxins fumonisin (FUM) and zearalenone (ZEN) was tested by the ELISA quantitative analysis method. The result revealed that FUM was produced only by *F. fujikuroi* and that ZEN was produced by *F. asiaticum* and *F. graminearum*.

## 1. Introduction

Herbal medicine is a kind of medicine that uses herbal products (plant products such as flowers, seeds, shrubs, tree branches, moss, lichens, seaweed, and algae and fungi including mushrooms). According to the World Health Organization, 80% of people living in developing countries mostly rely on herbal and/or traditional medicines [1], but herbal or alternative medicines are also popular in developed countries. China and India are the largest producers and users of herbal and traditional medicines. Barnes et al. [2] mentioned that about half of Australians and one third of Americans use alternative medicines. Korea produces and uses medicinal plants in different forms, and Korean adlay (*Coix lacrymal-jobi* L.) is an herbal plant used as a traditional medicine on the Korean peninsula. The plant grows in tropical, subtropical, and temperate regions and is popular in Korea, China, and Japan [3]. In Korea, seed production of adlay is the fifth highest among that of medicinal plants with an annual income of 26.5 billion won [4]. In comparison with other cereals, adlay contains high levels of protein, lipids, and fiber [5]; and, along with large amounts of calcium, iron, and vitamin B1, this makes the plant attractive as an alternative food source [6,7]. The seeds in a powder form are known to ameliorate the effect of some diseases. It has been claimed that adlay plant seeds could reduce the risk of cancer and lower blood cholesterol levels, and they produce coixol—a functional material of some medicines [8,9,10].

Adlay seeds are infected with different pathogens in the field and after harvest. *Fusarium* species (*F. graminearum*) have been reported as one of the most important pathogens from the plant [11,12]. *Fusarium* is one of the most important fungal genera that produces diseases on cereals and in the mycological taxonomy.

*Fusarium* species are capable of producing a wide variety of mycotoxins in pre-harvest infected plants in fields and in storehouses [13]. Mycotoxins pose a significant risk to human and animal health and are generally produced by five fungal genera: *Alternaria*, *Aspergillus*, *Claviceps*, *Fusarium*, and *Penicillium* [14]. *Fusarium* mycotoxins are important as they are disease causing agents of plants, animals, and human [15]. The *Fusarium* species that are the producers of mycotoxins include the *Fusarium oxysporum* species complex, *F. graminearum* species complex, *F. solani* species complex, *F. poae*, and *F. verticilliodes* [16].

The objective of this study is to examine the prevalence and molecular characterization (by combined analysis of two protein coding genes, EF-1α and β-tubulin) of *Fusarium* species in adlay (*Coix lacrymal-jobi* L.) seeds in Korea and to examine the main mycotoxin production ability of *Fusarium* species by ELISA quantitative analysis for fumonisin (FUM) and zearalenone (ZEN).

## 2. Results

### 2.1. Mycofloral Incidence

**Figure 1:**
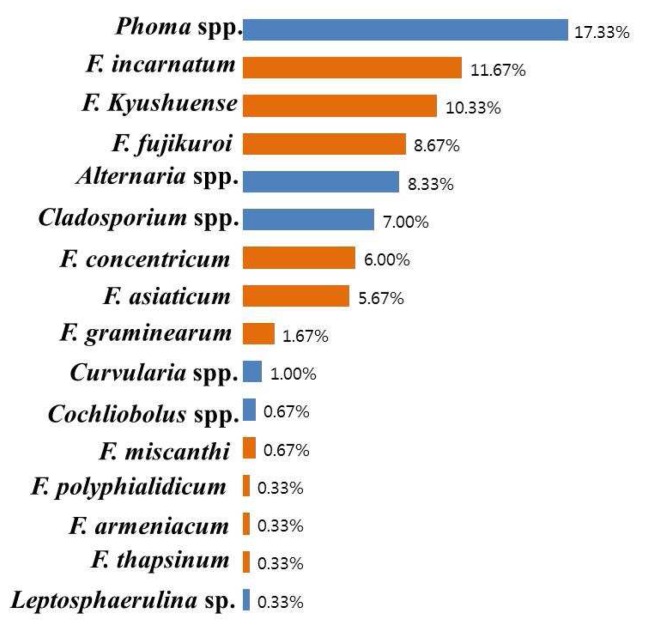
Percentage incidence of seed-borne fungi on adlay (400 seeds) based on morphology and ITS gene sequence analysis.

A total of 400 fungal isolates belonging to seven genera and 20 species were detected from adlay seeds by the standard blotter and test tube agar methods. The different fungal populations isolated from the medicinal plant were *Alternaria*, *Cladosporium*, *Cochliobolus*, *Curvularia*, *Fusarium*, *Leptosphaerulina*, and *Phoma* (Figure 1). Among the isolated fungi species, 45.6% were *Fusarium* species followed by *Phoma* (17.33%), *Alternaria* (8.33%), *Cladosporium* (7.00%), *Curvularia* (1.00%), *Cochliobolus* (0.67%), and *Leptosphaerulina* (0.33%).

### 2.2. Incidence of Fusarium

**Figure 2:**
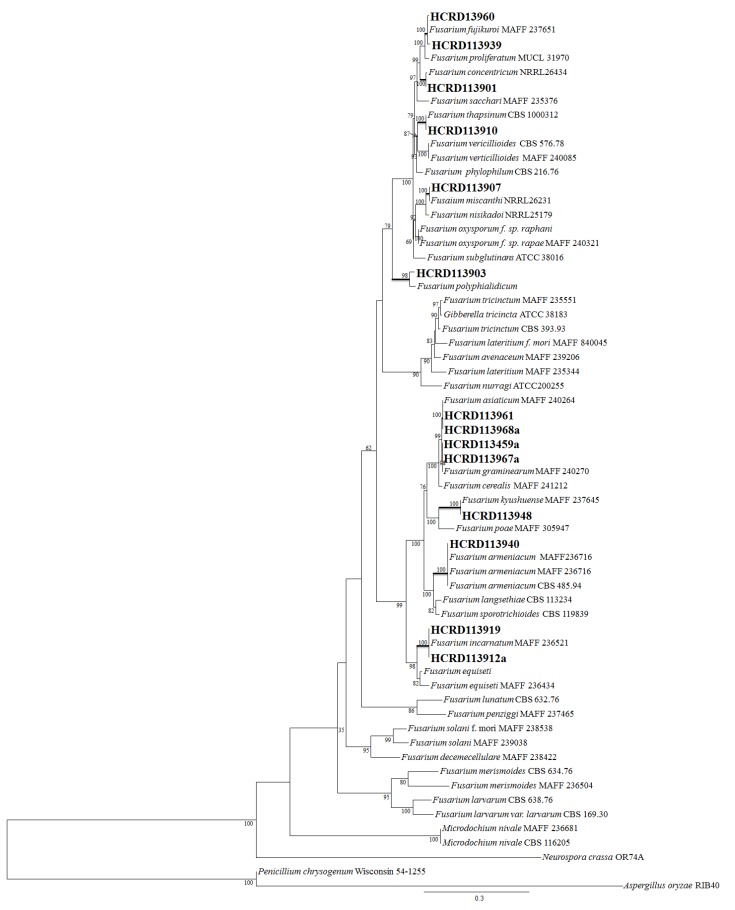
Phylogenetic relationship of *Fusarium* spp. Maximum likelihood tree of the *Fusarium* and related genera inferred from the combined sequences of the β-tubulin and elongation factor genes. Taking into account the different tempos and modes of nucleotide substitutions, all parameters of the substitution model were separately estimated for each gene using the GTR + I + Γ model. The branch lengths are proportional to the estimated number of nucleotide substitutions. The bootstrap probability (BP; 1000 replicates) values over 75% are displayed on the nodes. Fungi isolated from adlay seed are indicated in bold.

**Figure 3:**
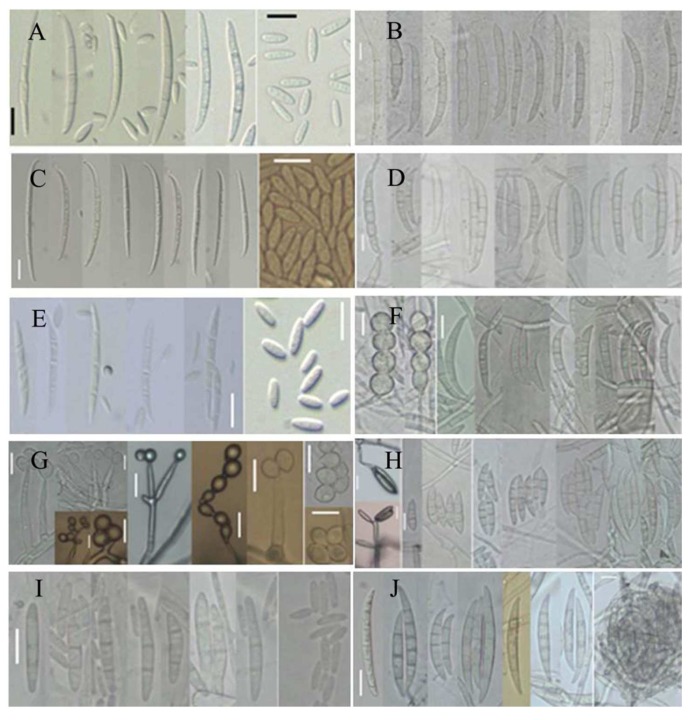
Ten species of *Fusarium* isolated from adlay. Scale bar = 10 μm: (**A**) *Fusarium fujikuroi*; (**B**) *Fusarium asiaticum*; (**C**) *Fusarium concentricum*; (**D**) *Fusarium graminearum*; (**E**) *Fusarium thapsinum*; (**F**) *Fusarium armeniacum*; (**G**) *Fusarium miscanthi*; (**H**) *Fusarium kyushuense*; (**I**) *Fusarium polyphialidicum*; and (**J**) *Fusarium incarnatum*.

The *Fusarium* species isolated were superficial fungi from adlay seeds, and they were divided into different groups. Among the different groups, representative isolates were selected and sequenced with different primers. Based on the internal transcribed spacer (ITS) region of the ribosomal DNA (rDNA) and phylogenetic analysis of elongation factor 1-alpha (EF-1α) and beta-tubulin (BT2) genes by the maximum likelihood (ML) method, 10 species of *Fusarium* were characterized (Figure 2). The identified fungal species and incidence percentages were *F. incarnatum* (11.67%), *F. kyushuense* (10.33%), *F. fujikuroi* (8.67%), *F. concentricum* (6.00%), *F. asiaticum* (5.67%), *F. graminearum* (1.67%), *F. miscanthi* (0.67%), *F. polyphialidicum* (0.33%), *F. armeniacum* (0.33%), and *F. thapsinum* (0.33%). Representative isolates were then grown on synthetic nutrient-poor agar (SNA), carnation leaf agar (CLA), and potato-dextrose agar (PDA) to examine morphological characteristics. Morphological studies of these representative fungi correlated well with the molecular analysis. The conidial characteristics of the 10 different *Fusarium* species are shown in Table 1 and Figure 3.

### 2.3. Occurance of Mycotoxin in Different Fusarium Species

**Figure 4:**
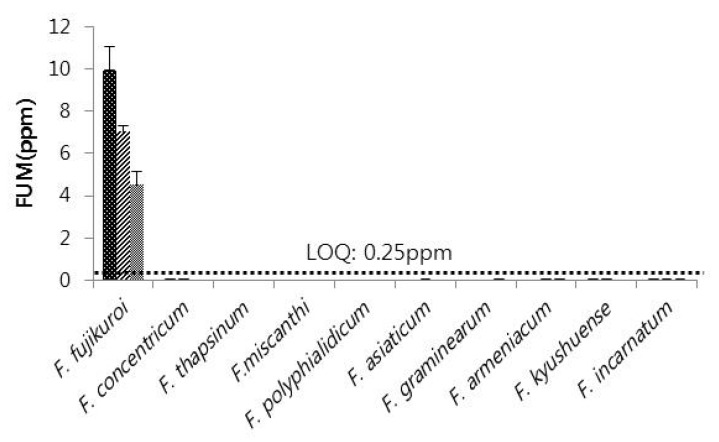
Fumonisin producing ability of ten *Fusarium* species isolated from adlay seeds. LOQ, limit of quantitation; FUM, Fumonisin.

**Figure 5:**
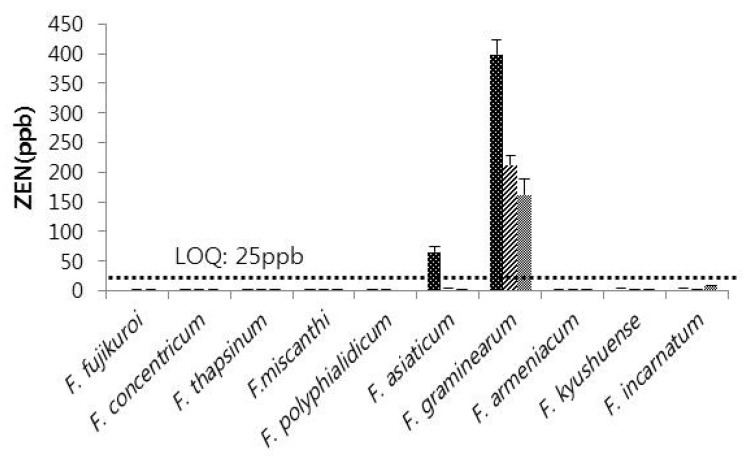
Zearalenone producing ability of ten *Fusarium* species isolated from adlay seeds. LOQ, limit of quantitation; ZEN, Zearalenone.

Collected *Fusarium* samples were investigated for toxin producing ability by ELISA quantitative analysis. The experiment was conducted three times with three replications from each single *Fusarium* isolate. The result confirmed that among the ten species of *Fusarium*, FUM production was highly associated with *F. fujikuroi* with none of the other species producing FUM. The average production of FUM in the three experiments was 9.92 ppm, 7.02 ppm, and 4.52 ppm (Figure 4). ZEN production was restricted to *F. asiaticum* and *F. graminearum* among the *Fusarium* species isolated from adlay seeds. Although *F. fujikuroi*, *F. asiaticum*, and *F. graminearum* produced FUM or ZEN, no detectable amounts of FUM and ZEN were produced by isolates of the other seven *Fusarium* species. *F. graminearum* produced relatively high amounts of ZEN (398.94 ppb, 212.53 ppb, and 161.85 ppb in three replications) compared with *F. asiaticum* (64.94 ppb) (Figure 5).

## 3. Discussion

Adlay (*Coix lacrymal-jobi* L.) is one of the most common medicinal resources. It also supplies foods as well as drinks (e.g., tea). This plant grows well in oriental countries, where traditional medicines are well known, popular, and consumed for medicinal purposes. In this study, adlay seeds showed a prevalence of diversified fungal flora with the predominant fungal genera being *Fusarium* (45.6%) and *Phoma* (17.33%). It has been shown that *Fusarium*, *Phoma*, *Alternaria*, *Penicillium*, *Aspergillus*, and *Cladosporium* spp. are the commonly isolated mycoflora from cereal seeds and other plant seeds [13,17,18,19,20]. Almost all the common species were detected except *Penicillium* and *Aspergillus*. These two fungi are most common in many seeds. A reason for their absence may be that competitive interactions exist among *Fusarium*, *Aspergillus*, and *Penicillium*. Studies revealing a negative correlation between *Fusarium* spp. and *Penicillium* spp. were reported in grains by Martin et al. [21] and Barros et al. [22]. Chen et al. [17] offered a similar explanation for the prevalence of *Fusarium* where *Aspergillus* and *Penicillium* were not recorded.

Among species found that are associated with seed diseases of adlay, *Fusarium* species predominated and accounted for 45.6% of total fungi isolated. In addition, this was the genus with the largest number of species with ten species of *Fusarium* characterized. *Fusarium* species are difficult to identify, and multilocus molecular data analysis along with morphological characterization have to be applied. Internal transcribed spacer (ITS) and phylogenetic analysis with two protein coding genes, elongation factor 1-alpha (EF 1α) and beta-tubulin (BT2) grouped the isolated *Fusarium* into 10 groups, each of different species. Herron et al. [16] recommended similar molecular characterization methods to identify *Fusarium*. In this study, morphological characterization perfectly correlated with the molecular data analysis. The 10 species recovered based on the two analytical methods were *F. incarnatum*, *F. kyushuense*, *F. fujikuroi*, *F. concentricum*, *F. asiaticum*, *F. graminearum*, *F. miscanthi*, *F. polyphialidicum*, *F. armeniacum*, and *F. thapsinum*. *F. incarnatum* and *F. kyushuense* accounted for 11.67% and 10.33%, respectively, of all *Fusarium* species isolated, while *F. fujikuroi*, *F. concentricum*, and *F. asiaticum* accounted for 8.67%, 6.00%, and 5.67%, respectively. *F. graminearum*, *F. miscanthi*, *F. polyphialidicum*, *F. armeniacum*, and *F. thapsinum* recorded relatively low figures of 1.67%, 0.67%, 0.33%, 0.33%, and 0.33%, respectively. Other research also found a number of *Fusarium* species. Bottalico et al. [13] encountered 15 species of *Fusarium* associated with head blight in small grain cereals in Europe. Chen et al. [17] claimed that 51% of fungi associated with spider flower seed were *F. incarnatum*. Eight of the most common *Fusarium* species were isolated from Norwegian cereals by Langseth et al. [18].

In this study, production of the toxins FUM and ZEN was investigated. Among all the species, only *Fusarium fujikuroi* was found to produce FUM, whereas only two of the species isolated produced ZEN, namely *F. asiaticum* and *F. graminearum*. In Korea, staple foods are monitored for toxins produced by *Fusarium*. In addition, barley and maize are checked for *Fusarium*, and *Fusarium* was found to occur with a frequency of 29.8%, 6.4%, and 36.2% in barley and 2.3%, 55.8%, and 34.9% in maize. Among all samples, FUM was only detected in one sample from corn and it had a permissible threshold level of 100.90 μg/kg [23]. The prevalence of *Fusarium* species and their ability to produce mycotoxins (zearalenone, moniliformin, and fumonisin B1) was examined in Zimbabwean corn; and it was observed that only one species produced all the three mycotoxins simultaneously, whilst most produced fumonisin B1 and/or moniliformin and only nine isolates produced ZEN [24]. In Europe, mycotoxin production by *Fusarium* was examined in head blight of small-grain cereals, and the study revealed that *F. graminearum* and *F. culmorum* produced the highest amounts of ZEN in north European areas [13]. A high occurrence of ZEN was described by Srobarova and Pavlova [25] in ears of winter wheat highly contaminated with *F. graminearum.* In Austria, kernel samples of durum wheat, predominantly infected by *F. graminearum* with a lower presence of *F. culmorum*, contained low levels of ZEN [26].

## 4. Conclusions

A small scale research effort has been conducted worldwide to characterize the mycobiota and toxigenic effect of fungi, especially *Fusarium* from medicinal plant seeds. In the oriental region (Korea, Japan, and China), medicinal plants and their tissues are valued greatly as traditional oriental medicine; therefore, the prevalence of fungi (with a special focus on *Fusarium*) on adlay seeds from Korea was investigated for the first time. Adlay is the host species of different fungal taxa including *Fusarium*, *Phoma*, *Alternaria*, *Cladosporium*, *Curvularia*, *Cochliobolus*, and *Leptosphaerulina.* Among the fungi detected in this study, *Fusarium* accounted for the greatest number and constituted 45.6% of the total. The collected *Fusarium* samples were investigated for their toxin producing ability by ELISA quantitative analysis, and the results showed that FUM was produced by *F. fujikuroi* and ZEN by *F. asiaticum* and *F. graminearum*.

## 5. Materials and Methods

### 5.1. Plant Species and Sample Collection

Adlay (*Coix lachrymal-jobi* L.) is commonly known as Job’s tears, but it is commonly sold as Chinese pearl barley in Asian supermarkets. Besides their use for ornamental purposes, adlay grains are useful as a source of cereal foods and traditional folk medicine. In Korea, adlay tea (yulmu cha) is a popular drink. The plant is grown in some parts of Korea. Adlay seeds were collected from 3 adlay growing regions (Yeoncheon, Hwasun and Eumseong) during 2012. Seed samples were picked and collected randomly from farmers’ fields, and 10 different locations were chosen from each adlay growing region. From each location around 50 gm of seeds were packed in sterile polyethylene bags and brought to the laboratory. Within 5 h of collection, the samples were processed for isolation of fungi.

### 5.2. Mycofloral Isolation and Analysis

**Figure 6:**
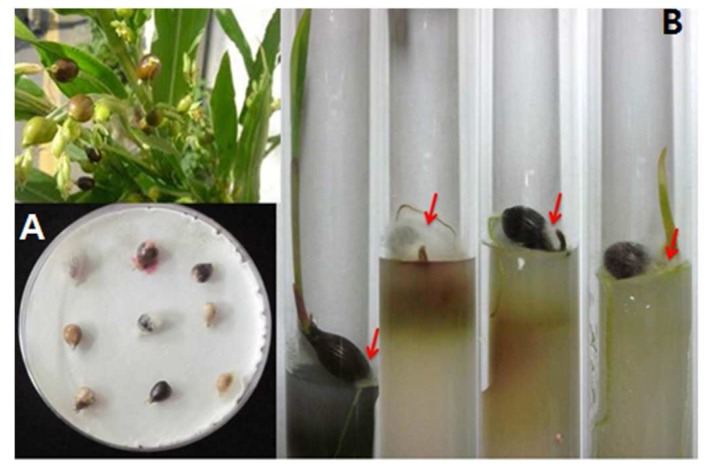
Incubation of adlay seeds: (**A**) The Blotter method, Adlay seed incubated for 7 days at 20 °C under 12/12 h NUV/dark cycle; and (**B**) Test tube agar method, Adlay seed incubated for 3 weeks at 20 °C under 12/12 h daylight/dark cycle.

Among all the samples collected, 400 seeds were tested for fungal occurrence by standard blotter and test tube agar methods. Seeds were surface sterilized by the methods described by Paul et al. [27]. For the blotter method, adlay seeds were incubated on wet filter paper for 7 days at 20 °C under a 12/12 h NUV/dark cycle; and, for the test tube agar method, seeds were incubated for 3 weeks on test tube poured with PDA media at 20 °C under a 12/12 h light/dark condition cycle (Figure 6). After 2 days of incubation on wet filter paper and after one week by the test tube agar method, seeds were checked under a stereomicroscope for fungal hyphae and conidial growth, and then seeds were checked on every subsequent day during the periods mentioned above. Hyphae and conidia were then transferred to PDA media and finally pure cultures were prepared. Pure cultures of isolates were maintained in PDA slant tubes and 20% glycerol stock solution and deposited in the culture collection of the Chungnam National University Fungal Herbarium and Rural Development Administration (RDA), Eumseong, Korea.

### 5.3. Molecular Identification of the Isolates

#### 5.3.1. DNA Extraction, PCR, and Purification

All the fungal isolates were grown on PDA for 7 days. Genomic DNA was extracted by the method described by Paul et al. [28]. The internal transcribed spacer (ITS) region of the ribosomal DNA (rDNA) was used in this study for PCR amplification of all isolates [29]. The ribosomal DNA of the *Fusarium* isolates was then separated, and PCR amplification was carried out with that of two genes—elongation factor 1-alpha (EF 1α) and beta-tubulin (BT2) (Table 2). The amplification reaction for each gene was performed in 50 μL reaction volume and carried out in a GeneAmp PCR System 2700 thermo cycler (Applied Biosystems, Foster City, CA, USA), under conditions described by Deng et al. [30] (Table 3). The Wizard PCR prep. kit (Promega, Madison, WI, USA) was used for purification of successfully amplified PCR products. Sequencing of strands was performed with an ABI Prism 310 Genetic Analyzer (Applied Biosystems, Foster City, CA, USA) using a BigDye Terminator Cycle Sequencing Kit (Applied Biosystems) with the same primer used for PCR amplification.

#### 5.3.2. Sequencing and Phylogenetic Analysis

The obtained sequences and other sequences retrieved from the previous study or in GenBank were initially aligned with the CLUSTAL X (v 2.0, 2007, the Conway Institute UCD Dublin, Dublin, Ireland) program [31], were edited in BioEdit (v 7.0.1, Tom Hall, 2013, Ibis Biosciences, CA, USA), and were completed with manual adjustment. For the combined analysis, the genes were concatenated in a single nucleotide alignment. Maximum likelihood analysis was conducted using RAxML (v 7.2.8 HPC, The Exelixis Lab 2013, Scientific Computing Group, Heidelberg, Germany) by employing the GTRGAMMA model of nucleotide substitution. The robustness of the phylogram in the maximum likelihood analyses was evaluated by 1000 bootstrap replications. The best tree obtained from this search was edited in Mega (v 5.05, 2011, The Biodesign Institute, AZ, USA) [32]. 

### 5.4. Analysis of Mycotoxins

#### 5.4.1. *Fusarium* Inoculation for ELISA

Adlay seeds were dehulled and 10 g of seeds was mixed with 5 mL sterilized distilled water in a 50 mL tube. Tubes were then autoclaved for 30 min at 121 °C. After cooling, 5–6 mycelial plugs (6 mm) of 5-day cultured *Fusarium* species on PDA media were transferred to the tube. Tubes were incubated for 30 days at 15 °C. Before toxin analysis, all the samples were dried in a dryer at 60 °C until the weight of each sample again reached 10 g again and then the samples were stored at −20 °C prior to the toxin analysis.

#### 5.4.2. Sample Preparation and Toxin Analysis

To analyze and quantify levels of FUM and ZEN in adlay, enzyme-linked immunosorbent assay (ELISA) was performed using the commercial kits- AgraQuant Fumonisin Test Kit and AgraQuant Zearalenone Test Kit (Romer Labs GmbH, Tulln, Austria). Sample preparation and analysis were done according to manufacturer instructions written in the handbook. All samples were ground to a fine powder, whereby over 70% of the powder passed through a 0.5 mm mesh sieve. For FUM and ZEN analysis, prepared samples of 10 g were mixed with 50 mL of distilled water and then homogenized in a Waring blender at high speed for 3 min. Extracts were filtered through Whatman filter paper No. 1 (Whatman, Maidstone, UK).

### 5.5. Morphological Characterization of Toxigenic Fusarium

*Fusarium* species isolated from adlay seeds were grown on potato dextrose agar (PDA; Difco, Montreal, QC, Canada) at 20 °C in the dark for description of aerial mycelium and pigmentation. Colony diameters were measured after 7 days of incubation and other characteristics such as texture, color, and pigmentation were also recorded. For description of conidial morphology, isolates were grown on synthetic nutrient-poor agar (SNA) beneath fluorescent lights (12/12 light/dark) at 20 °C to induce sporulation. Randomly selected conidia (50) from 7-day-old cultures were used to obtain conidial measurements and photographed using an OLYMPUS BX50 light microscope (OLYMPUS, Tokyo, Japan) with an Artcam 300 MI digital camera (ARTRAY, Tokyo, Japan). Morphological characteristics of the isolate were then compared with previous descriptions.

## Figures and Tables

**Table 1:** Comparison of morphological characteristics of ten different *Fusarium* species.

Structure		Characteristics *									
		Ff	Fc	Ft	Fm	Fp	Fa	Fg	Far	Fk	Fi
Macroconidia	Septa	3	3–5	3	3–5	3	3–5	5–6	2–3	3–5	3–5
	Size	38–41.1 × 3.26–3.24	33.08–45 × 2.5–2.75	21–29 × 2.5–2.7	40–57.2 × 3.8–4.1	27.87–30.1 × 2.8–3	39.63–60.5 × 4.69–6.22	54.7–65.2 × 4–5	25.5–27 × 3.5–4	31–41 × 4–5.5	23–36 × 3–4
	Shape	Relatively slender	Relatively slender with no significant curvature	Relatively slender, slightly falcate or straight	Relatively slender	Relatively wide, straight, stout and robust	Curved to straight	Curved to straight with ventral surface	Prominently curved	Falcate to fusiform	Relatively slender with a curved dorsal surface
Microconidia	Septa	0–1	0–1	0	0	0–2	-	-	-	0–3	0–1
	Size	6.48–16.19 × 1.14–2.27	6.14–9.36 × 2.29–2.6	6.2–7 × 2–2.5	7.5–11 × 5–7	8.76–16.71 × 2.46–4.51	-	-	-	18.7–20 × 2–4	4.5–9.6 × 2–3
	Shape	Oval, club shaped with a flattened base	Oval, obovoid to allantoid	Club shaped with a flattened base	Pyriform	Fusiform or subclavate	-	-	-	Fusiform to falcate	Fusiform

* Ff, *Fusarium fujikuroi*; Fc, *F. concentricum*; Ft, *F. thapsinum*; Fm, *F. miscanthi*; Fp, *F. polyphialidicum*; Fa, *F. asiaticum*; Fg, *F. graminearum*; Far, *F. armeniacum*; Fk, *F. kyushuense*; Fi, *F. incarnatum*.

**Table 2:** Nucleotide sequences of primer sets used for amplifying target genes.

Primers	Primer Sequences	References
ITS5	GGAAGTAAAAGTCGTAACAAGG	White et al. 1990
ITS4	TCCTCCGCTTATTGATATGC	
EF1	ATGGGTAAGGAAGACAAGAC	O’Donnell et al. 2000
EF2	GGAAGTACCAGTGATCATGTT	
EF3	GTAAGGAGGASAAGACTCACC	
EF2T	GGAAGTACCAGTGATCATGTT	
Btu-F-F01	CAGACCGGTCAGTGCGTAA	Watanabe et al. 2011
Btu-F-R01	TTGGGGTCGAACATCTGCT	

**Table 3:** PCR condition for amplifying target genes used in this study.

Gene	Initial Denaturing	Denaturing	Annealing	Extension	Final Extension	Cycle
ITS (ITS5, ITS4)	94 °C, 10 m	94 °C, 30 s	55 °C, 30 s	72 °C, 1 m	72 °C, 10 m	30
EF (EF1, EF2)	94 °C, 5 m	94 °C, 30 s	52 °C, 40 s	72 °C, 1 m	72 °C, 3 m	35
EF (EF3, EF2T)	94 °C, 5 m	94 °C, 30 s	53 °C, 30 s	72 °C, 1 m	72 °C, 5 m	40
β-tubulin (BT2a, BT2b)	94 °C, 5 m	94 °C, 30 s	60 °C, 30 s	72 °C, 1 m	72 °C, 3 m	35
